# The Potential Impact of Intelligent Systems for Mobile Health Self-Management Support: Monte Carlo Simulations of Text Message Support for Medication Adherence

**DOI:** 10.1007/s12160-014-9634-7

**Published:** 2014-08-01

**Authors:** John D. Piette, Karen B. Farris, Sean Newman, Larry An, Jeremy Sussman, Satinder Singh

**Affiliations:** 1 Center for Clinical Management Research, VA Ann Arbor Healthcare System, Ann Arbor, MI USA; 2 Department of Health Behavior and Health Education, University of Michigan School of Public Health, Ann Arbor, MI USA; 3 Department of Internal Medicine, University of Michigan Medical School, Ann Arbor, MI USA; 4 Department of Social and Administrative Services, College of Pharmacy, University of Michigan, Ann Arbor, MI USA; 5 Division of Computer Science and Engineering, Department of Electrical Engineering and Computer Science, College of Engineering, University of Michigan, Ann Arbor, MI USA

**Keywords:** Medication adherence, Mobile health, Text messaging, Simulation models, Chronic disease management

## Abstract

**Background:**

Mobile health (mHealth) services cannot easily adapt to users’ unique needs.

**Purpose:**

We used simulations of text messaging (SMS) for improving medication adherence to demonstrate benefits of interventions using reinforcement learning (RL).

**Methods:**

We used Monte Carlo simulations to estimate the relative impact of an intervention using RL to adapt SMS adherence support messages in order to more effectively address each non-adherent patient’s adherence barriers, e.g., forgetfulness versus side effect concerns. SMS messages were assumed to improve adherence only when they matched the barriers for that patient. Baseline adherence and the impact of matching messages were estimated from literature review. RL-SMS was compared in common scenarios to simple reminders, random messages, and standard tailoring.

**Results:**

RL could produce a 5–14 % absolute improvement in adherence compared to current approaches. When adherence barriers are not accurately reported, RL can recognize which barriers are relevant for which patients. When barriers change, RL can adjust message targeting. RL can detect when messages are sent too frequently causing burnout.

**Conclusions:**

RL systems could make mHealth services more effective.

**Electronic supplementary material:**

The online version of this article (doi:10.1007/s12160-014-9634-7) contains supplementary material, which is available to authorized users.

## Introduction

Patient non-adherence to medications for chronic disease management is a major cause of preventable morbidity, mortality, and excess healthcare spending. As many as half of all chronically ill patients fail to take their medications as prescribed, contributing to nearly 100,000 premature deaths annually, as well as $290 billion in annual healthcare costs [1–3] and more than a 10 % increase in hospital admissions for older adults [4].

While forgetfulness is one major challenge to medication use, non-adherence often is intentional, due to patients’ skepticism about the medication’s potential benefits, or concerns regarding side effects or other negative consequences of long-term pharmacotherapy [5–7]. Patients often report multiple reasons for not taking their medication as prescribed [5]. Those reasons differ among patients and can change over time as patients’ health beliefs are influenced by factors such as their receipt of health information or changes in their health status [8, 9].

Mobile health (mHealth) interventions such as patient text messaging (short message service or SMS) can deliver frequent prompts and health information to improve adherence [10–16]. Two large trials in Kenya have shown that SMS messages can improve antiretroviral adherence among patients with HIV/AIDS, with one of those trials demonstrating significant improvements in viral suppression [17, 18]. Studies from other countries also demonstrate that SMS messaging may improve medication adherence [13–16]. Some of the most successful text messaging adherence interventions have focused on medication reminders [19].

One well-established, evidence-based technique to ensure that patients using mHealth support services receive the information they need is to tailor messages according to the characteristics of the user [20]. Most studies of tailored health communication use comprehensive baseline assessments to determine what information will be most impactful for which patients [20], typically focusing on patients’ sociodemographic characteristics, clinical history, health beliefs, and other determinants of self-care behavior. Message tailoring can significantly improve the effectiveness of health behavior change messages, including messages addressing medication underuse [21–23].

Despite its demonstrated benefits, the effectiveness of tailored communication may be limited if the assessments used to tailor information fail to address key determinants of users’ behavior, or if valid patient information cannot be collected due to psychometric limitations in the measures or biases in patient reports. For some behaviors, such as medication adherence, accurate data are often lacking about what causes patients to fall short of self-care goals [24]. Patients’ need for health information may change over time as they master skills or develop new concerns, and repeated tailoring to accommodate those changes is challenging. Finally, studies have shown repeatedly that patients can become desensitized to health communication [25–28], and current approaches to tailored adherence support typically cannot detect patient burnout or adapt message content and frequency so that services remain engaging and effective. The next generation of chronic disease behavior change services will need new strategies for building on the successes of tailored messaging based on up-to-date evidence regarding the content and mode of communication that are most effective for each individual.

Reinforcement learning (RL) is a field of artificial intelligence that presents new opportunities to automatically adapt mobile health communication based on feedback from patients about the impact of different types of messages with respect to attaining a given health or behavioral goal. RL algorithms applied to mobile health communication for adherence support can use information about the effectiveness of prior messages addressing each patient’s reasons for non-adherence while continuing to strategically explore patients’ responses to messages addressing other potential adherence challenges that have yet to be identified. RL has been applied extensively in robotics, control systems, and resource allocation tasks [29, 30], and RL algorithms are common in digital commerce, including web advertisement; online news article selection; or product recommendation by companies such as Google [31], Yahoo [32], Amazon, and Netflix. Applications of RL to human-centered tasks related to behavior change are rare [33–35], and to our knowledge, only a few published papers have described the use of RL in mobile health disease management support [36].

The purpose of this study was to demonstrate the ways in which RL could be used to adapt the content and frequency of SMS messages to promote adherence among individuals taking a medication regularly for a chronic medical problem. To that end, we conducted Monte Carlo simulations estimating the impact of an RL-driven SMS adherence support tool relative to other common approaches to designing mobile health messaging systems, such as focusing specifically on patient reminders or tailoring communication using a baseline survey. Monte Carlo simulations allow potential impacts of a healthcare intervention or policy to be evaluated without the costs of service development and deployment, patient enrollment, and outcome measurement in a real-world trial [37–39]. A strength of simulations is that intervention effects can be estimated in the context of differing scenarios (e.g., whether or not patients misreport their adherence barriers, making standard tailoring more difficult) as well as in the context of random variability in key parameters, such as patients’ daily adherence behavior. Through the simulations presented here, we estimated the relative performance of RL compared to three common approaches to mobile health messaging and in the context of real-world challenges to the effectiveness of SMS behavior change services, including a change in patients’ adherence barriers over time and decreased intervention effectiveness when some patients become desensitized to messages that are sent too frequently.

## Methods

### Components of a Reinforcement Learning System for SMS Adherence Support

An RL system is composed of four functional units: (1) a set of action choices (in this case, the types of SMS messages addressing various potential reasons for non-adherence); (2) a means of obtaining feedback or “reinforcement” about the outcome of interest following each action choice, i.e., the “reward” in RL terminology; in the case of medication use, the reward can be defined as an ongoing measure of medication adherence during successive intervals; (3) Additional information about the “state” or context within which action choices are made, such as patients’ characteristics affecting adherence as well as information about patients’ prior history of interaction with the system; and (4) an RL computational agent (the RL “engine”) that adapts probabilistic action choices based on the state, action, and reward data that are accumulated over time. Each of these components is described below. Additional technical details about RL are included in the Electronic Supplementary Material (ESM).

#### Action Choices

Action choices represent the set of potential interventions available to the RL system at each time point. For the simulations described here, we defined action choices as the choice to send an SMS message addressing one of three possible reasons for medication non-adherence. Specifically, we reviewed the literature [6, 40, 41] and identified three common barriers to appropriate medication use: (1) patients’ beliefs about their disease and its implications for their health and well-being, e.g., beliefs that their illness is not serious or that their risk for complications is minimal; (2) patients’ beliefs about their medication, e.g., that the medication cannot change their long-term course of illness or that it will cause side effects; (3) patients’ need for strategies to address forgetfulness in taking their medication. In the simulations, the RL system was designed to “learn” for each patient which message type or action was most effective in promoting adherence; and (4) in the third simulation below, examining the impact of message fatigue, a null message (i.e., not sending any message), was considered a fourth action choice so that the system could learn the frequency of messaging that was most effective for each patient.

#### Adaption Based on Feedback

RL systems adapt by seeking to optimize a numerical outcome or “reward.” Operationally, an SMS medication adherence support system using RL would need a “sensor” for collecting data about patients’ medication taking following each message sent to the patient as well as a mathematical model for translating those adherence data into a score that the system uses as its reward to evaluate the impact of those action choices. A sensor could be something simple, such as an SMS system with which patients could repeatedly self-report their adherence using a numerical rating scale. For the simulations presented here, we assumed that the RL system would be informed about patients’ adherence using daily pill bottle openings provided automatically via a medication electronic monitoring system (MEMS) [42–45].

#### State Information

When making action choices, some RL algorithms can take into account baseline information about each patient as well as information gathered during the history of interaction between the patient and the system. Such “state” information summarizes what is important to action selection. State parameters can be thought of as “effect modifiers” that influence the expected impact of a given action on patients’ adherence. In SMS intervention design, state information could include information such as changes in adherence after sending previous messages, adherence barriers or health beliefs reported at baseline, or the patient’s demographic characteristics. RL systems learn the best mathematical function relating state variables with probabilistic action choices. By learning an action-selection policy from prior interactions with users and applying that policy to future interactions with patients who share similar characteristics as recorded in their vector of state information, RL systems can learn more quickly how best to target messages so as to maximize the impact on the “reward.”

#### The RL Engine

There are several types of RL algorithms that could be used to process reward data and drive SMS adherence support message-selection choices [29, 46–48]. Regardless of this choice, all RL systems must address two fundamental problems: (1) balancing exploration of relatively untested action choices versus exploitation of information about choices that have already been shown to be impactful and (2) accounting for delayed effects of a given action choice on patients’ future adherence behavior. This latter problem is particularly relevant in mobile health services supporting behaviors such as medication adherence, since information from SMS adherence messages hopefully affects patients’ adherence not only the day the message was received but also the patient’s long-term ability to self-manage their chronic disease. One subset of RL approaches (known as “bandit” algorithms) assumes that an SMS message sent today influences only the patient’s likelihood of adherence that same day, i.e., the message will have no residual impact on their adherence in the future. Other algorithms (e.g., algorithms based on partially observable Markov decision processes or POMDP’s) estimate a parametric model for patients’ underlying pattern of behavior and use that knowledge to account for long-term effects. These differences between algorithms can contribute to the speed with which an RL system can effectively adapt to each patient’s unique needs (i.e., the number of patients and patient interactions needed to optimize message choices) as well as the maximum impact that the system may have across patients and over time. Parametric models can be slow to learn, while algorithms that learn quickly may not have the greatest long-term effect. In the simulations that follow, we used a contextual bandit algorithm, LinUCB (see ESM for more information), to illustrate the way in which an RL-supported SMS adherence program can adapt quickly to each patient’s needs and have a relatively important impact even in the absence of a parametric model of patients’ adherence behavior [49].

### Monte Carlo Simulations

To evaluate the performance of RL-based adherence messaging relative to other approaches, we conducted three Monte Carlo simulations. For each simulation, we assumed that patients’ adherence behavior was determined by the extent to which they experienced three adherence barriers, represented by the three “*α*_(*barrier*)_” terms in the following formula:

$$ P\left(\mathrm{adherence}\right)={\alpha}_{\left(\mathrm{disease}\right)}\times {\alpha}_{\left(\mathrm{medicine}\right)}\times {\alpha}_{\left(\mathrm{remember}\right)} $$

where *P*(adherence) is the probability that a patient takes his or her medication correctly each day, *α*_(disease)_ represents the extent to which the patient believes that his or her illness is important to treat (ranging from values of 0 for not at all important to 1 for extremely important), *α*_(*medicine*)_ represents the extent to which the patient is concerned about the negative consequences of his or her medication use (ranging from 0 for extremely concerned to 1 for not at all concerned), and *α*_(*remember*)_ represents the likelihood that the patient remembers to take his or her medication on a given day (ranging from 0 for will not remember to 1 for will definitely remember).

For example, if a patient is only 80 % convinced about the importance of treating his or her illness (i.e., *α*_(disease)_ = 0.80), the patient is only 70 % concerned about taking the medication (i.e., *α*_(medicine)_ = 0.70), and if the patient has only a 90 % probability of remembering to take the medication assuming he or she intends to do so (i.e., *α*_(remember)_ = 0.90), then that patient’s probability of taking medication on a given day would be as follows:

$$ (1): P\left(\mathrm{adherence}\right)=0.80\times 0.70\times 0.90=0.504 $$

In the simulations presented here, we assumed that the patient sample included 20 patients whose only adherence barrier was a doubt about the disease’s severity, 20 patients whose only barrier was a concern about the safety and efficacy of the medication, and 20 patients who both had doubts about disease severity as well as problems with forgetfulness. Based on a literature review [4, 5, 8, 9, 50, 51], we assumed that the value of each *α*_(barrier)_ in our simulations was 0.65. Studies for medications treating hypercholesterolemia, osteoarthritis, cardiovascular disease, and type 2 diabetes report adherence rates ranging from 50 to 72 % [3, 51]. Other studies including conditions such as bladder disorders and glaucoma have found adherence rates below 40 % [2]. Given that all three *α*_(barrier)_ terms = 0.65, the baseline adherence rate for the overall sample of simulated patients was in this range, i.e., 57 % or the average of 65 % for the 20 patients with only doubts about their disease’s severity, 65 % for the 20 patients with only medication belief concerns, and 42.25 % for the 20 patients with both disease belief as well as forgetfulness problems. Patients’ actual adherence on a given day was assumed to vary around their own average adherence level, according to random variation in the relevant *α*_(barrier)_ terms, with each day’s value for each patient drawn from a Gaussian distribution with a mean of 0.65 and a standard deviation of 0.30.

SMS messages were assumed to impact adherence only if they addressed the patient’s underlying reasons for non-adherence as expressed by that person’s three *α*_(*barrier*)_ values. For example, we assumed that a reminder message would have no impact if the patient in fact was not forgetful but rather was non-adherent because of unaddressed concerns about the medication’s safety. When a given SMS message did address one of the patient’s reasons for non-adherence, the magnitude of the SMS message effect or “β_(match)_” was calculated as a multiplicative improvement in the relevant *α*_(barrier)_. For example, if β_(match)_ = 0.6 and a patient only had medication concerns with *α*_(medicine)_ = .65, then their *α*_(medicine)_ would change to the following:

$$ (2):{\alpha}_{\left(\mathrm{medicine}\right)}=0.65+0.6\times \left(1.0-0.65\right)=0.86 $$

In the absence of an empirical evidence for differences in the effect size of SMS messages addressing each of the three adherence barriers, we assumed that the effect of a matching SMS message was the same for all three. The magnitude of β_(match)_ used in the simulations presented below was based on the improvement in adherence reported in SMS adherence trials, i.e., 12 to 16 % [17, 21, 50, 51]. For example, Pop-Eleches et al. [17] found that weekly short reminders could improve adherence by up to 13 % over the control group after 48 weeks, and Petrie et al. [21] showed that text messages tailored to patient’s underlying adherence barriers could improve adherence up to 15 % over a control group. To ensure that our simulations generated results that were comparable to these outcomes, we assumed that each day, the actual value of β_(match)_ for each patient was selected at random from a normal distribution with a center of 0.7 and a standard deviation of 0.3.

**Fig. 1:**
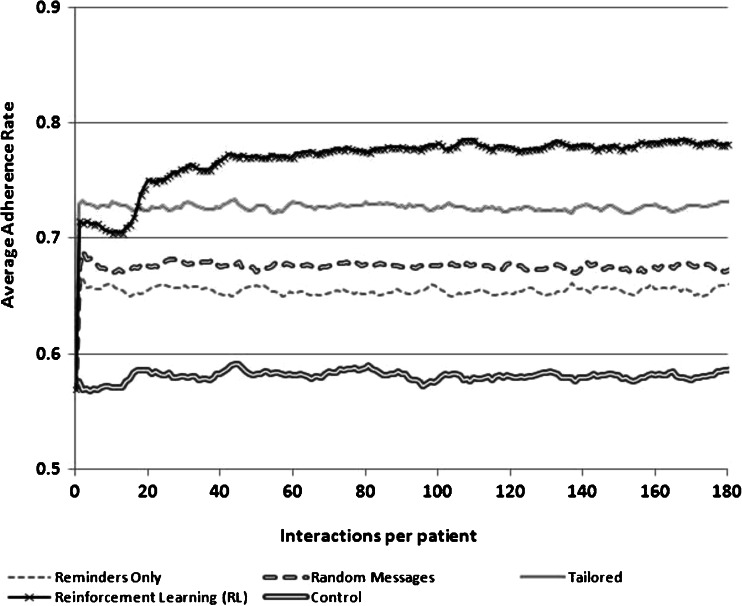
Adherence rates achieved using four different message-selection strategies when a third of the population does not accurately self-report their non-adherence barrier

**Fig. 2:**
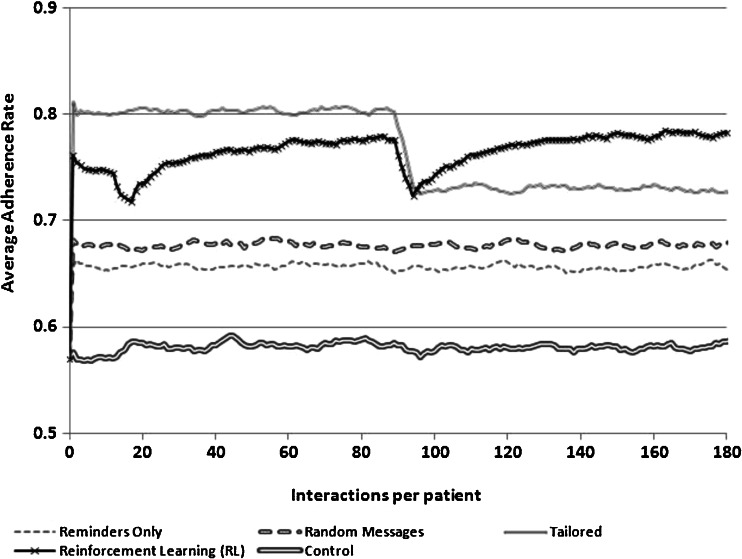
Adherence rates achieved using four different message-selection strategies when a third of the population change their non-adherence barrier on day 90

**Fig. 3:**
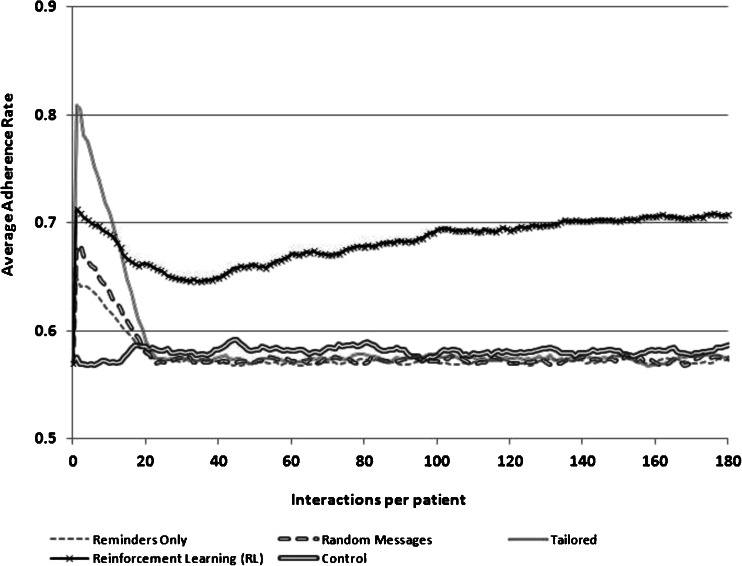
Adherence rates achieved using four different message-selection strategies when there is message fatigue

Given these assumptions, the goal of the RL-supported messaging system was to increase the intervention’s effectiveness over time via an increased likelihood of a match between the type of message sent to each patient on each day and the patient’s adherence barriers. RL sought to increase the match success rate by modifying the probability of sending a message addressing each of the three barriers, based on feedback about that patient’s adherence following prior choices by the RL engine as to which message type would be most effective. More details about the characteristics of each simulation are presented in Table 1 and ESM. The results of the simulations are presented graphically in Figs. 1, 2, and 3 as changes in the proportion of patients who took their medication each day over the course of a 180-day intervention period. Estimates for each day of adherence support were averaged over 100 independent runs, with key parameters being drawn from Gaussian distributions, as noted above.

**Table 1:** Summary of simulation experiments

	Simulation 1	Simulation 2	Simulation 3
Aim	Estimate the relative performance of an RL agent assuming that a third of the sample does not accurately report their non-adherence barrier at baseline	Estimate the relative performance of an RL agent assuming that a third of patients’ underlying barrier to adherence changes during the course of intervention	Estimate the relative performance of an RL agent assuming that patients “tune out” messages that come too frequently
Action choices	1. Send a disease belief message 2. Send a medication belief message 3. Send a remembering strategy message	1. Send a disease belief message 2. Send a medication belief message 3. Send a remembering strategy message	1. Send a disease belief message 2. Send a medication belief message 3. Send a remembering strategy message 4. Send no message to learn about the impact of message frequency
State variables	How many times the patient took his/her medication following the last 5 times a message of each type was sent		1. How many times the patient took his/her medication following the last 5 times a message of each type was sent 2. A binary indicator for whether a message had been sent on each of the previous 2 days
Adherence after 180 days^a^	RL: 78 % (0.5 %) Tailored: 73 % (0.6 %) Random: 67 % (0.5 %) Reminders: 66 % (0.6 %)	RL: 78 % (0.6 %) Tailored: 73 % (0.6 %) Random: 68 % (0.6 %) Reminders: 66 % (0.5)	RL: 70 % (0.6 %) Tailored: 57 % (0.6 %) Random: 57 % (0.6 %) Reminders: 56 % (0.7 %)

For each experiment, we assumed that the population consisted of 60 patients including the following: 20 patients whose only barrier to medication adherence was concerns about the safety and efficacy of their medications, 20 patients with doubts about their disease severity, and 20 patients who had both concerns about their medications as well as problems remembering to take them

*RL* reinforcement learning

^a^The baseline adherence rate for the overall sample was assumed to be 57 %, based on a literature review. Numbers in parentheses are standard deviations

We compared the behavior of the RL-SMS adherence support service to three common alternatives to delivering SMS messaging interventions: (1) SMS messages serving only as medication adherence reminders, (2) SMS messages addressing each of the three potential barriers to adherence but sent randomly so that each patient received all message types on random days, and (3) SMS messages tailored to patients’ adherence barriers as reported in a baseline survey. The RL algorithm initially weighted its choice of messages according to the baseline information about the patient’s barriers—similar to the tailored approach. However, unlike tailoring, message choices for RL were selected from a probability distribution in which messages addressing other potential adherence barriers were selected with a small probability, so that RL could “explore” these choices, learn from subsequent adherence feedback, and adjust message targeting accordingly.

The relative efficacy of the RL-based messaging system was evaluated in three scenarios, representing possible situations that could limit the efficacy of current approaches: (1) a scenario in which some patients’ actual barriers to medication adherence are not accurately measured at baseline, (2) a scenario in which some patients’ reasons for non-adherence change during the course of intervention, and (3) a scenario in which patients experiences SMS message fatigue and begin to tune-out messages that come too frequently. *In the first scenario*, we assumed that patients who were actually concerned about their medication’s safety instead reported at baseline that their problem was forgetfulness. *In the second scenario*, we examined what would happen when patients who originally had doubts about their medication’s safety instead developed doubts about their disease severity roughly halfway through the intervention period. In each of these two scenarios, we assumed that patients received SMS messages daily.

*In the third scenario*, we assumed that patients experienced message burnout when they received messages too frequently and that “too frequently” meant that they received a message after having received a message on each of the two previous days. For patients experiencing message burnout, we used a multiplicative fatigue factor that diminished the daily effect of a well-targeted message (recall that mismatched messages were assumed to have no effect). Specifically, we assumed that the effect of a given SMS message matching the patient’s adherence barrier decreased 5 % each day that the patient was fatigued. For patients in the three non-RL comparison groups, we assumed that the SMS system continued to send messages daily despite message fatigue. In contrast, in the RL-supported program, the system was designed to “learn” automatically to adapt to message burnout by evaluating the impact of sending one of the three message types versus sending no message on a given day to prevent message fatigue. Each day that the patient was not fatigued, the impact of a well-matched message increased 10 % until it reached its maximum level. See the ESM for more details.

## Results

### Simulation #1: Adaptation to Misreported Reasons for Non-Adherence

As shown in Fig. 1, in the initial 10 days of messaging, a tailored system resulted in higher rates of improvement in medication adherence than the alternative messaging approaches. During that initial period, the RL system (lacking experience from which to learn) began by randomly sending some messages to patients and therefore matched their adherence support needs less frequently than the tailored approach. However, with greater experience, the RL system was able to target messages effectively according to their impact on each patient’s actual adherence, i.e., regardless of the patient’s reported reasons for non-adherence at baseline. In contrast, the tailored approach continued to send mismatched messages to simulated patients who reported forgetfulness at baseline but who actually had concerns about their medication’s safety. As a consequence, the overall improvement in adherence for the tailored system was less than for the RL system at 180 days.

The simple reminders performed worse than all other messaging strategies because only one third of the simulated sample had forgetfulness as an adherence problem, and those patients also had concerns about their disease severity. Random messaging performed slightly better than simple reminders, since each message had at least a one third chance of being relevant for a particular patient. For simulated patients with both disease belief and memory barriers, each random message had a two thirds chance of being relevant. At the end of 180 days, the estimated daily adherence rate was 78 % for the RL messaging system, 73 % for the tailored system, 67 % for random messaging, and 66 % for simple reminders.

### Simulation #2: Changes Over Time in Patients’ Reasons for Non-Adherence

When assuming that simulated patients did in fact accurately report their reasons for non-adherence at baseline, the tailored approach, because it was immediately able to match adherence support messages to patients’ needs, exceeded all other approaches in the initial period of intervention and continued to do so for the first 90 days (Fig. 2). During that period, the RL approach progressively improved relative to random messaging and simple reminders but still performed worse than tailored messages. However, when simulated patients who originally had concerns about their medication’s safety developed doubts about their disease severity halfway through the intervention period, the tailored approach experienced a significant drop in effectiveness for users who thereafter had a mismatch between their message tailoring and their actual need for adherence support. The RL approach also experienced a drop in effectiveness at 90 days, since “lessons learned” in the first 90 days led the system to send messages incorrectly to patients whose adherence barriers changed. However, as shown in Fig. 2, the RL system was able to adapt to the change in patients’ adherence barriers and began to outperform the tailored approach as well as the other two approaches around day 100. At the end of 180 days, expected daily adherence rates were 78 % for the RL system, 73 % for tailored messaging, 68 % for random messages, and 66 % for simple reminders.

### Simulation #3: The Impact of Message Fatigue

As shown in Fig. 3, message fatigue led to diminishing intervention impact among patients receiving tailored messages, random messages, or simple reminders. This is because these messaging strategies were unable to adapt to the fact that when fatigued, the impact of well-targeted messages was progressively less. This resulted in a steady drop in the adherence rate for the three comparison groups, until the adherence rate in each group reached the baseline rate. In contrast, the RL messaging strategy was able to learn that the highest adherence rate for these simulated patients was achieved by repeating the pattern of sending two consecutive well-targeted messages followed by not sending a message on the third day. Initially, the adherence rate in the RL messaging group declined as the RL system learned how patients responded (and in the process, elicited fatigue in some patients). However, by 20 interactions per patient, the adherence rate in the RL group started rising. At the end of 180 days, the expected daily adherence rate was 70 % for the RL system while the comparison groups returned to their baseline adherence rate of 57 %.

## Discussion

We used Monte Carlo simulations and information about the effectiveness of adherence support services from prior trials to estimate the impact of an RL-based SMS adherence support tool compared to other common approaches to SMS adherence messaging. Our results indicate that RL is able to learn to personalize messages to individuals based on their true underlying causes for non-adherence, adapt to changes in the underlying causes for non-adherence over time, and adapt the frequency of messaging so as to avoid message fatigue. The RL system was able to outperform current approaches to designing SMS adherence interventions given reasonable assumptions regarding each approach’s effectiveness when SMS messages matched patients’ adherence barriers and assuming a relatively small sample of patients. These results affirm our underlying hypothesis that RL-based systems can overcome some of the limitations of current approaches to mHealth service design including approaches that tailor health communication via fixed information collected about patients’ health beliefs at baseline. RL systems learn from increasing experience. As a consequence, systems will become increasingly effective as the number of patients and patient interactions grows. Because mHealth interventions can be expensive to create but less expensive to maintain, they become more efficient in the context of larger sample sizes, and therefore, RL may be ideally suited to situations in which mHealth programs are likely to be deployed.

It is important to emphasize that the scenarios explored in the current simulation trials are common in chronic disease management. For example, it is well known that patients often misreport their adherence barriers [19], and time-pressed clinicians may not be able to administer extensive tailoring surveys at program entry. For both of these reasons, RL services that require little tailoring information at baseline are an attractive alternative. Moreover, changes in patients’ health status and adherence barriers are the hallmark of chronic illness, and researchers have had difficulty identifying services that can continue to be engaging and relevant in order to maintain health benefits over the long-term. Because RL-based behavior change services continually adapt according to what is working for each patient, they represent a more patient-centered and potentially more effective alternative to current approaches.

“Message fatigue” is a major problem in chronic disease management [25–28]. Fatigue has not been addressed adequately because tolerance for frequent messages likely varies across patients, and addressing fatigue means balancing that problem with the need to make interventions as intensive as possible. Unlike less flexible approaches to messaging, RL can use feedback from patients to develop schedules that meet each individual’s unique needs and preferences for information.

Our simulations leave open a number of challenging questions. Consistent with the underlying motivation for an adaptive approach, generalizations about the impact of RL behavioral interventions relative to standard messaging strategies are likely to be of limited relevance for a given application, due to the diversity of: the clinical problems for which an adaptive intervention might be useful, the characteristics of patients with whom these interventions could be applied, and the action choices that could be available to the RL system. Multiple empirical studies will be needed addressing a wide range of health behavior challenges to fully understand where and to what degree RL systems can improve behaviors over-and-above current, deterministic interventions.

Nevertheless, some general conclusions can be drawn about the characteristics of RL-based interventions that are likely to lead to a more successful adaptation to patients’ needs and therefore greater effectiveness. Action choices need to be defined so that they represent distinct and meaningful alternatives that behavioral theory suggests may have varying, measurable impacts on outcomes across patients and over time. To the extent possible, the “reward” signal should be reliable, tightly linked to the action choices made by the RL system, and reported frequently enough to allow for rapid and successful intervention adaptation. Here, we presented a hypothetical example in which reward data consisted of daily adherence information available from a MEMS cap. Of course, MEMS cap information is rarely available in real-world settings and advances in passive sensor technologies may expand the number of situations in which RL-based interventions are successful in promoting behavior change. Even without those advances, researchers should continue to identify instances in which patients’ self-reports can be used to guide adaptation by RL systems, since reliable behavioral reports that are correlated in predictable ways with the outcome of interest may be adequate, even if those self-reports are biased relative to the actual behavior, e.g., over-reporting of medication taking.

The mathematics needed to know how to “power” clinical trials for RL-based systems is underdeveloped relative to what is known for more standard fixed arms trials. The use of simulations, as in this paper, is one approach to determining how many patients are needed to learn a good RL policy. The benefit of these simulations depends on the availability of good mathematical models of patient behavior in response to RL system actions as well as good models of noise in the reward signal. If such models are available, then simulations can be used to fine tune the parameters of the RL algorithm (and more generally select from among competing RL algorithms) as well as determine how many interactions with patients are needed for the system to effectively learn what actions are most effective for each user. Further simulation-based and theoretical work is needed to articulate these important relationships as they apply to real-world applications, such as SMS adherence support.

Some of the parameters we used were based on real data (e.g., the baseline adherence rates and the expected impact of a message addressing a patient’s adherence barriers), while other parameters such as the distribution of adherence barriers in the population were more arbitrary. To some extent, the results of our simulations reflect these choices. For example, a third of the sample in the current simulations had both concerns about their medication as well as problems remembering to take it; if those patients only had problems with forgetfulness, then random messages (with a third of those messages being reminders) would have had roughly the same impact on adherence in the overall sample as messaging exclusively targeting forgetfulness. If baseline adherence rates were significantly higher than the 57 % estimate we used, RL would have less opportunity to improve over standard tailoring. In contrast, if baseline adherence rates were much lower, then the differences between RL and simple tailoring would be larger than that shown in Figs. 1, 2, and 3. Auxiliary analyses that we conducted varying these assumptions as well as others such as the proportion of patients who misreport their adherence barriers demonstrate that the *relative* performance of RL compared to current mHealth messaging strategies would be largely unaffected by these differences. Nevertheless, the true benefits of an adaptive, patient-centered approach will be situation-specific, and more research should be pursued to understand where and how to best apply this new approach to enhancing health behavior change.

Studies have shown repeatedly that improved adherence is linked with improved health. For example, investigators have reported that hypertensive patients who are “non-adherent” have a 3.8-fold risk of stroke-related death in 2 years [52], 43 % of “high adherence” patients with hypertension achieve blood pressure control compared to 33 % of those with “medium adherence” [53], and “lower adherers” to statins have a 25 % increased risk of death compared to “high adherers” [54]. The precise functional form of the relationship between adherence and health is not well described, and it is difficult to generalize based on these simulations and available literature about specific health benefits that could be achieved through a more adaptive approach to behavioral intervention design. Nevertheless, it is fair to say that the absolute improvements in adherence shown here relative to tailored messaging are modest, suggesting that the details of intervention design as well as the population in which that intervention is evaluated will be crucial determinants of effectiveness in real-world studies of RL-based adherence support systems. For example, auxiliary analyses indicate that if the baseline adherence rate were only 32 %, a third of the population did not accurately report their adherence barrier, and if a third developed a new adherence barrier, the difference in adherence with RL versus standard tailoring would be more than twice that presented here.

In summary, this simulation study demonstrated the possible benefits of RL in mHealth communication for the improvement of medication adherence. We found that under many real-world circumstances, RL could be more effective than tailored messages, because RL systems can learn the needs of each individual patient and how those needs change with time. RL systems also may provide a solution to patient burnout, by adapting the frequency of messages to each user so that they meet the person’s needs and preferences. RL-based mHealth interventions are a promising example of how artificial intelligence can make healthcare more patient-centered.

## Electronic Supplementary Material

Below is the link to the electronic supplementary material.

ESM 1 (DOC 108 kb)
