# Supplementary material for: The Potential Impact of Intelligent Systems for Mobile Health Self-Management Support: Monte Carlo Simulations of Text Message Support for Medication Adherence
Source: Ann Behav Med. 2014 Aug 1;49(1):84–94. doi: 10.1007/s12160-014-9634-7 (PMC4335096; doi:10.1007/s12160-014-9634-7)
Supplement: Supplementary file 1 — (DOC 108 kb) [file 12160_2014_9634_MOESM1_ESM.doc]

**ELECTRONIC SUPPLEMENTARY MATERIAL**

**The Potential Impact of Intelligent Systems for Mobile Health Self-Management Support:**

**Monte Carlo Simulations of Text Message Support for Medication Adherence**

**Overview of Reinforcement Learning and LinUCB**

Reinforcement learning (RL) is a field of artificial intelligence that allows an “intelligent agent” to learn what treatment choices work best in order to optimize a measurable outcome (termed the system’s “reward”; see Figure 1).

The process used to optimize treatment choices in RL mimics the way that humans learn skills such as riding a bicycle, i.e., through behavioral experiments, systematic adaptation and generalization, and targeted trials of new behaviors with measurable outcomes. RL algorithms similar to those we illustrate in this study are the basis of online consumer targeting programs such as Netflix, Google, and Amazon.com,[^1^](#_ENREF_1) where a service learns automatically how to deliver information that is most relevant to each user. Introductory texts and papers describing RL and LinUCB have been published previously.[^2-7^](#_ENREF_2)

| 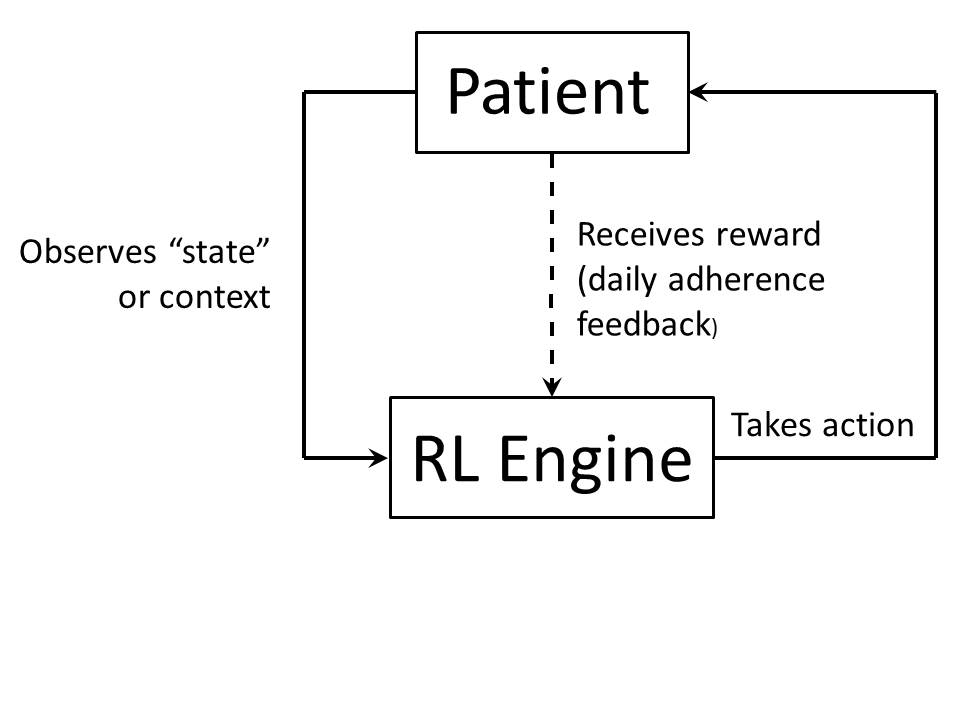 |
| --- |
| **Figure 1. The Reinforcement Learning feedback loop.** The RL action choices in the current Monte Carlo simulation were SMS messages sent each day addressing three potential reasons for non-adherence. For the third simulation, the RL engine also explored a fourth action choice, i.e., to not send any message in order to explore the impact of message fatigue. |

In the simplest k-armed bandit framework, an agent is presented with a choice of *k*-arms (in the current simulations, the arms are choices of message types to send) where each arm has an initial unknown reward distribution associated with it (here, the probability of medication-taking after receipt of each message type). The agent’s goal is to maximize the expected reward (i.e., maximize expected medication-taking). This leads to the exploration-exploitation problem where exploration involves learning more about the expected reward of each possible action choice, while exploitation involves repeatedly selecting the action choice that experience to date suggests has the highest expected reward. Too little exploration may lead the RL agent to not discover the best choice and consequently not gain as much reward as it could have, while too much exploration will result in the agent too often selecting the action that historically has not had the highest payoff.

In the “contextual bandit” approach used in these simulations, the RL agent uses state data or contextual information in addition to information about the outcome for the most recent action choice. Here the agent's goal is to choose the best action given a particular context. In our example, state data could be a feature vector containing information about a patient's demographic characteristics, health beliefs, or clinical status. State data also could include information about the person’s pattern of responses to previous message selections. Thus, in addition to allowing action choices to depend on how an individual patient has been responding in the past and therefore allowing RL to personalize its behavior for each patient, state data allow message type selection policies learned from one patient to be generalized to other patients who share the same context, as recorded in their state vector. By doing so, RL can use experience more effectively and adapt more quickly for new patients who have similar characteristics as those it has encountered in the past.

In summary, the LinUCB algorithm combines two ideas in order to solve the exploration-exploitation problem. First, it estimates the expected reward of an action as a linear function of context (hence Lin) via incremental regression, and second it estimates and uses upper-confidence-bounds (hence UCB) for each action choice in a context to add a kind of reward-bonus to the current expected reward estimate. This has been shown to provably and effectively solve the exploration-exploitation tradeoff.

**Simulating the Impact of RL-Supported SMS Adherence Support**

In simulations 1- 2, the objective for the RL agent was to choose each day the action (i.e., send either a disease message, a medication belief message, or a remembering strategy message) that would maximize the reward signal (modeled as daily pill-bottle openings). Simulation 3 in which we considered the effect of message fatigue included a fourth action choice of not sending a message. We further assumed that each patient’s adherence behavior could be characterized by the following formula:

| *P(adherence) = α_(disease)_ * α_(medicine)_ * α_(remember)_* |
| --- |
| Where: *P(adherence)* is the patient’s probability of taking his or her medication each day; *α_(disease)_* is the extent to which the patient sees his or her illness as important to treat (ranging from values of 0 for not at all important to 1 for extremely important); *α_(medicine)_* represents the extent to which the patient is concerned about the negative consequences of their medication use (ranging from 0 for extremely concerned to 1 for not at all concerned); and *α_(remember)_* is the probability that the patient remembers to take his or her medication on a particular day (ranging from 0 for will not remember to 1 for will definitely remember). |

For example, if a patient is only 80% convinced about the importance of treating his or her chronic disease, i.e., *α_(disease)_* = 0.80, and the patient is 70% concerned about taking the medication, i.e., *α_(medicine)_* = 0.70, and the patient has only a 90% probability of remembering to take the medication assuming s/he intends to do so, i.e., *α_(remember)_* = 0.90, then that patient’s likelihood of taking his or her medication on a given day would be:

(1): *P(adherence)* = 0.80 * 0.70 * 0.90 = 0.504.

**The Impact of SMS Messages on Adherence.** We assumed that SMS messages only had an impact if they addressed the patient’s underlying reasons for non-adherence. For example, we assumed that a reminder message would have no impact if the patient in fact was concerned only about the safety of his or her medication use. Each day the impact of an SMS message addressing one of the patient’s reasons for non-adherence, we assumed that β*_(match)_* could be characterized as a multiplicative change in that component of the formula determining the patient’s adherence probability. For example, if β*_(match)_* = 0.6, and the patient represented by (1), above, received a message addressing his or her medication concerns, then the coefficient representing the magnitude of those concerns would change from 0.70 to:

(2): *α_(medicine)_* = 0.70 + 0.6 * (1.0-0.70) = 0.88,

and the patient’s overall probability of adherence would increase from 0.504 to:

(3): *P(adherence)* = 0.80 * 0.88 * 0.90 = 0.6336.

In the simulations, we assumed that β*_(match)_* was the same for each underlying non-adherence cause and for each patient. Research shows that SMS adherence interventions have a range of impacts on patients’ baseline adherence rate. SMS reminder interventions have shown effects ranging from no significant improvement up to improvements of 12% to 16% over control groups not receiving SMS reminders.[^8-10^](#_ENREF_8) The duration of these studies ranged from 3 months to 12 months. Additionally, a tailored intervention[^11^](#_ENREF_11) showed an average difference of 15% between the adherence rate of the intervention group receiving tailored SMS messages and that of a control group over the course of 9 months. From these results, we derived a β*_(match)_* effect of 0.7 because this yields improvements in the population’s adherence rate roughly in line with these prior studies’ findings. This value of β*_(match)_* results in an increase of 7% for reminders only and 14% for tailored messaging, with the assumption that a third of the population misreport their adherence barrier. An improvement of approximately 23% would have been possible for tailored messaging under our assumptions, if all patients accurately self-reported their adherence barrier. Because our RL system is adaptable, we assumed that RL based messaging would be able to achieve an improvement that was on the high side of this range (21%).

**Message Fatigue.** In Simulation 3, we introduced two long-term effects of messaging. The first long-term effect is that of message fatigue, i.e., a reduction in β*_(match)_* among patients who receive too many messages and begin to ignore them. Specifically, we assumed that patients become fatigued if they receive a message after having received a message on each of the previous two days. In contrast, patients are assumed to be not fatigued on any day that this condition does not hold. Fatigue reduces the effect of messages, and this reduction becomes more accentuated with each additional day of fatigue. Specifically, the first time patients get fatigued we assumed that their β*_(match)_* drops to 0.95*β*_(match)_* where 0.95 is the *fatigue factor*. For example, for the patient represented in formula (1), above, when the patient is first fatigued, *α_(medicine)_* would instead be:

(4): *α_(medicine)_ = α_(medicine)_ + fatigue factor ** β*_(match)_* * (1 – *α_(medicine)_*) *= 0.7 + 0.95 ** 0.6 * 0.3 = 0.871

And the adherence rate would be

(5): *P(adherence)* = 0.80 * 0.871 * 0.90 = 0.627

instead of the 0.6336 shown in formula (3). The fatigue factor becomes 5% worse each day that the patient remains fatigued. If the patient continues to get messages every day, this results in the patient returning to their baseline adherence rate as they tune out the messages. On the other hand, every day the patient is not fatigued, the fatigue factor is lessened by 5%. Thus, after several days of not getting fatigued, the patient recovers in that well-targeted messages have their full effect.

The second long-term effect of messaging introduced in Simulation 3 is that well-targeted messages keep their effect beyond the day they are sent. Specifically, if a patient receives two consecutive well-targeted messages (messages that address the patient’s underlying adherence barriers), then if that patient does not receive a message on the next day, he or she still experiences 50% of the effect of a well-targeted message (i.e. 0.5*β*_(match)_*)*.* This effect only lasts for one day. As an example of this lingering effect, if after receiving two consecutive medication concern messages, a patient receives no message on the third day, using the formula from (4), the *α_(medicine)_* would be:

(6): *α_(medicine)_ = α_(medicine)_ + lingering effect ** β*_(match)_* * (1 – *α_(medicine)_) = 0.7 + 0.5 ** 0.6 * 0.3 = 0.79,

and the adherence rate would instead be:

(7): *P(adherence)* = 0.80 * 0.79 * 0.90~ 0.569.

In contrast, if we had not included this lingering effect, the patient's adherence rate upon not receiving a message would be the baseline value of 0.5. These two assumptions of the long-term effect of messaging make the patient-model in Simulation 3 more realistic than the patient-models used in Simulations 1 and 2. By design, the best strategy for communicating with these more complex patients is to avoid getting the patient fatigued, by repeatedly sending two consecutive well-targeted messages followed by not sending a message.

**References**
